# Preoperative Serum Glycan Levels Reflect Progression of Patients With Hepatocellular Carcinoma

**DOI:** 10.1002/cam4.70285

**Published:** 2024-10-09

**Authors:** Sheng‐Sheng Liu, Lei Ye, Qing‐Qing Dai, Yi Gao, Guang‐Hou Chen, Hong‐Chuan Zhao, Wei‐Dong Du

**Affiliations:** ^1^ Department of Pathology The First Affiliated Hospital Anhui Medical University Hefei China; ^2^ Department of Neurosurgery The First Affiliated Hospital, Anhui Medical University Hefei China; ^3^ Department of Hepato‐Biliary‐Pancreas Surgery The First Affiliated Hospital, Anhui Medical University Hefei China; ^4^ Department of Pathology Anhui Medical University Hefei China

**Keywords:** biomarker, Hepatocellular carcinoma (HCC), prognosis, prognostic factor

## Abstract

**Background:**

Abnormal glycosylation is associated with tumors. The clinical value of serum glycans in assessing progression of hepatocellular carcinoma (HCC) patients remains a challenge.

**Methods:**

A study dynamically comparing levels of fifteen lectin‐specific glycans between preoperative and postoperative serum of 65 HCC patients was conducted via lectin biochip technology. Multivariable logistic regression analysis was used to address associations between serum glycan levels and clinicopathological characteristics. Kaplan–Meier analysis was used to evaluate the impacts of serum glycan levels on overall survival (OS) and progression‐free survival (PFS) of the HCC patients.

**Results:**

HCC patients presented significantly higher levels of the lectin‐specific glycans in preoperative serum than disease‐free individuals (*p* < 0.001 − *p* = 0.029), except ConA. The glycans in preoperative sera were significantly related to tumor size, pTNM, metastasis, BCLC stage, portal hypertension (PHT), and platelet count (PLT), respectively (*p* < 0.05). Multivariate logistic analyses indicated that tumor size and pTNM independently impact on glycan‐specific lectins either LTL, UEA‐I, VVL, NPL, WGA, PNA, MAL‐I, SNA, or PHA‐L (*p* = 0.003 − *p* = 0.044); BCLC stage and PLT were independent factors influencing the serum glycans recognizable DSA (*p* = 0.024) and SNA (*p* = 0.050), respectively. Surgical excision of tumor mass significantly reduced glycan levels in sera. Tumor differentiation, albumin, and ABO type significantly revealed independent influence on glycan‐specific lectins, such as RCA‐I (*p* = 0.024), VVL (*p* = 0.024), and Con A (*p* = 0.026) in the postoperative serum. HCC patients with high levels of VVL‐binding glycans significantly benefited from a longer OS time (*p* = 0.016, HR: 0.460, 95% CI: 0.237–0.892) and a better PFS time (*p* = 0.004; HR: 0.435, 95% CI: 0.237–0.799), respectively.

**Conclusion:**

Serum glycans could reflect surgical outcomes in at‐risk patients and become valuable biomarkers in evaluating the progression of HCC patients.

## Graphical abstract

To assess clinical values of serum glycan changes in patients with HCC, in this study, we dynamically measured levels of fifteen glycan‐recognizable lectins in the preoperative and postoperative serum of 65 HCC patients by a well‐established lectin biochip. We analyzed associations of serum levels of the lectin‐binding glycans and clinicopathological characteristics and overall survival (OS) and progression‐free survival (PFS) of the HCC patients. We concluded that measurement of glycan‐specific lectins was a promising blood‐based test for HCC. Serum glycans could reflect surgical outcomes in at‐risk patients and become valuable biomarkers in evaluating progression of HCC patients.

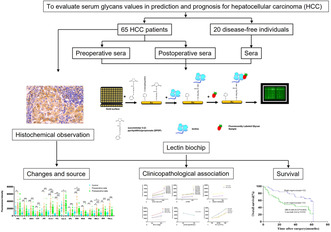

## 1 Introduction

Liver cancer is the third leading cause of cancer mortality worldwide, accounting for approximately 8.3% of all cancer death [1]. Hepatocellular carcinoma (HCC) contributed to 80% of total liver cancer cases [2]. Tumor bypasses cellular division checkpoints to evade death signals and immune surveillance, contributing to the development and metastasis of tumors. Glycan is one of the elementary structural components that confer protein versatility in biological function, immunogenicity, and cell recognition [3, 4, 5]. Investigations on glycosylation in health and diseases are well reviewed [4, 5, 6], highlighting that abnormal status of glycosylation is particularly associated with invasion and metastasis of tumors [4, 7, 8, 9]. Although detailed mechanisms of glycosylation for tumor progression have not been well elucidated, increased evidence indicates that tumor‐associated glycans are able to modulate anti‐tumoral immune response [10, 11] and to prevent cancer cells from recognition and eradication by the immune system [12]. Serum is a pool rich in glycoproteins and contains a variety of glycan structures, such as terminal fucose, sialic acid, mannose, galactose, N‐acetylgalactosamine (GalNAc), N‐acetyl‐D‐glucosamine (GlcNAc), and bi/tri/tetra antennary glycans [6]. It was reported that there were elevated fucosylation, sialylation, mannosylation, galactosylation, and/or branched glycans in HCC tissues [13, 14, 15] and enhanced fucosylation and sialylation in serum of patients with HCC [16]. Some glycosylated proteins have been developed as serum‐based biomarkers for HCC [5, 15, 17, 18]. Our previous meta‐analysis indicated that a combined assay of serum glycosylated proteins, such as AFP, AFP‐L3, DCP, GP73, and DKK‐1, was of impressive values in clinical decision‐making for HCC [19]. Thus, investigations on glycans profiling not only offer a new concept on tumor development but also provide novel insight into machinery study on surveillance and progression of HCC. However, unlike genomic or proteomic biomarkers, verifying glycans lacks biological template, and analysis for glycosylation models in HCC remains of great challenge.

Lectin is a category of carbohydrate‐binding protein, being able specifically to recognize unique motifs of glycans based on subtle structural differences. Lectin microarray enables rapidly analyzing glycosylation patterns of specific glycoproteins in biological samples and identifying glycoprotein biomarkers for cancer detection in vivo or in vitro [20, 21, 22]. A lectin‐based microarray investigation in patients with HCC and HBV infection revealed that expression of T/Tn antigen recognized by Jacalin (JAC) was significantly increased in both HCC and cirrhosis, and expressions of Galβ1‐3GalNAcα‐Ser/Thr and T antigens recognized by Peanut agglutinin (PNA) were markedly reduced in HCC tissues [23]. The evidence indicated that glycans might play potential roles in pathogenesis and progression of HCC. However, investigations on dynamic comparison of serum glycans and clinicopathological characteristics in patients with HCC have remained far overlooked. In this study, we performed a cohort study on changes of lectin‐specific glycans in preoperative and postoperative serum of HCC patients using a well‐established biochip platform. We analyzed associations between lectin‐specific glycan expressions and aggressive progression of HCC and evaluated potential predictive and prognostic values of serum glycans in HCC patients.

## 2 Materials and Methods

### 2.1 Reagents

Lectins, including Aleuria aurantia lectin (AAL, L‐1390‐2), Lotus tetragonolobus lectin (LTL, L‐1320‐5), Ulex europaeus agglutinin (UEA‐I, L‐1060‐2), Lens culinaris agglutinin (LCA, L‐1040‐10), Jacalin (JAC, L‐1150‐25), Ricinus communis agglutinin I (RCA‐I, L‐1080‐10), Vicia villosa lectin (VVL, L‐1230‐5), Concanavalin A (ConA, L‐1000‐500), Narcissus pseudonarcissus lectin (NPL, L‐1370‐5), Datura stramonium agglutinin (DSA, L‐1180‐5), Wheat germ agglutinin (WGA, L‐1020‐10), PNA (L‐1070‐5), Maackia amurensis lectin I (MAL‐I, L‐1310‐5), Sambucus nigra agglutinin (SNA, L‐1300‐5), Phaseolus vulgaris Leucoagglutinin (PHA‐L, L‐1110‐5), and biotinylated NPL (B‐1375‐2) were purchased from Vector Laboratories Inc. (CA, USA). Dimethyl sulfoxide (DMSO, 41640), bovine albumin (BSA, B2064), proteo Prep immunoaffinity albumin and IgG depletion kit (PROTIA), phosphate‐buffered saline (PBS, P3813), and 2‐[4‐(2‐hydroxyethyl) piperazin‐1‐yl] ethanesulfonic acid (HEPES, V900479) were commercially obtained from Sigma (MO, USA). Succinimidyl 3‐(2‐pyridyldithio) propionate (SPDP, 21857) was supplied by Thermo Scientific (MA, USA). Cy3 (AAT‐141) was purchased from AAT Bioquest (CA, USA). PD MiniTrap G‐25 column (28918007) was supplied from GE (MD, USA). Streptavidin peroxidase complex (KIT‐9710) and DAB kit (DAB‐0031) were purchased from MXB Biotechnologies (Fujian, China).

### 2.2 Clinical Samples

Sixty‐five patients with HCC were enrolled. Mean age was 57.05 ± 10.66 years old (range 30–77), including 57 men and 8 women. The patients underwent surgical operations in the Department of Hepato‐Biliary‐Pancreas Surgery, First Affiliated Hospital of Anhui Medical University of China between January 2018 and June 2019. All patients were pathologically diagnosed with primary HCC. Patients with other liver diseases, autoimmune diseases, infectious lesions of viruses and bacteria, other malignant tumors, and patients under chemotherapy before or after the surgery were excluded. Pairwise sera from the patients were collected before operation and on day seven after excision of tumor mass. In addition, 20 serum samples from disease‐free individuals, including 14 males and 6 females, with a mean age of 61.20 ± 7.42 years were collected. Table S1 showed clinical and pathological characteristics of the patients with HCC, including gender, age, blood type, body mass index (BMI), alcohol intake, smoking, albumin, total bilirubin, platelet count (PLT), alanine transaminase (ALT), aspartate aminotransferase (AST), α‐fetoprotein (AFP), hepatitis B surface antigen (HBsAg), liver cirrhosis (LC), portal hypertension (PHT), tumor location, tumor size, differentiation, lymphovascular invasion (LVI), capsule invasion, distant metastasis, pTNM. Liver function of the patients was evaluated based on Child‐Turcotte‐Pugh (CTP) score. Clinicopathological characteristics of patients were classified according to the eighth edition of the TNM staging standard for HCC of American Joint Committee on Cancer (AJCC) [24]. Clinical staging was determined with Barcelona Clinic Liver Cancer (BCLC stage) [25]. Clinical characteristics of patients with HCC are listed in Table S1. We performed a long‐term follow‐up study for the HCC patients up to 62 months. The mean survival time was 38.44 months. The median OS time was 47.00 months. The median PFS time was 29 months. Written consent from patients was obtained in the study. The study protocol conforms to the ethical guidelines of the Declarations of Helsinki and Istanbul as reflected in a priori approval by the Ethics Review Committee of Anhui Medical University.

### 2.3 Profiling Serum Glycans in HCC Patients With Lectin Biochip Technology and Lectin Histochemistry

Table S2 displayed fifteen lectins, including AAL, LTL, UEA‐I, LCA, JAC, RCA‐I, VVL, ConA, NPL, DSA, WGA, PNA, MAL‐I, SNA, and PHA‐L, recognizable glycans and potential indications. The lectins specifically recognize terminal glycotopes of fucose, α‐mannose, fucose, mannose, Galan, GalNAc, GlcNAc, sialic acid, branching and tri/tetra‐antennary complex oligosaccharides structures, respectively [26, 27]. Protocol for fabrication of SPDP‐modified biochip was described as our previous study [28]. Biochip measurement of serum glycans was performed according to our previous protocol [29]. In brief, serum samples were individually purified by ProteoPrep immunoaffinity Albumin/IgG Depletion Kit according to the manufacturer's instruction, to minimize potential interference of non‐specific glycoproteins in the serum. The purified sera were quantified with BCA Protein Assay kit, further labeled with an equivalent volume of Cy3‐NHS (1:1 Vol), and incubated in a dark environment at RT for 1 h. Labeled serum glycoproteins were separated from the excess free dye in the labeled reaction pool of sera by PD MiniTrap G‐25 column. Cy3‐labeled sera were obtained.

Each lectin was diluted in 10 mM HEPES buffer (pH 8.5)‐0.001% BSA to the concentration of 1 mg/mL, respectively, and was individually spotted onto SPDP‐modified biochips. The biochips were incubated in a moisture chamber at room temperature (RT) for 2 h to enable the lectins to efficiently immobilize. The Cy3‐labeled sera from 65 HCC patients and 20 healthy controls were individually loaded onto the lectin‐probed biochips and incubated in a moisture‐dark environment at RT for 1 h. After rinsed in PBST buffer twice at RT for 3 min and dried with a nitrogen flow, the biochips were scanned with LuxScan 10K/A scanner system (CapitalBio, Beijing, China). The background‐subtracted means value of each spot was recorded [30].

Histochemical staining for NPL in HCC and healthy hepatic tissues was described in our previous investigation [31]. In brief, the deparaffinized 4‐μm thick sections were incubated with biotinylated NPL at 0.01 M PBS buffer (pH 7.4)‐diluted concentration of 1:200 at RT for 30 min. After incubation with a streptavidin–peroxidase complex at RT for 10 min, the sections were visualized with DAB kit and counterstained with hematoxylin.

### 2.4 Statistical Analysis

The statistical analyses were performed by SPSS Statistics (version 23.0, IBM). Data were presented as the mean ± SD unless indicated otherwise. Continuous data were analyzed using Mann–Whitney *U* test or Kruskal–Wallis H test, followed by a multivariable logistic regression analysis. Correlation efficiency analysis was performed using Spearman's test. Diagnostic values for lectins in HCC were evaluated using receiver operating characteristic (ROC) curve. The cut‐off values were determined using the Youden index from the ROC curve. Survey for overall survival (OS) and progression‐free survival (PFS) were performed using the Kaplan–Meier analysis and log‐rank test. Cox proportional hazards regression was used for univariate analysis of prognosis. The *p* values reported in this study were two‐sided and *p* < 0.05 was significant.

## 3 Results

### 3.1 Level Changes of Lectin‐Specific Glycans in Sera of HCC Patients Before and After Operation

**FIGURE 1:**
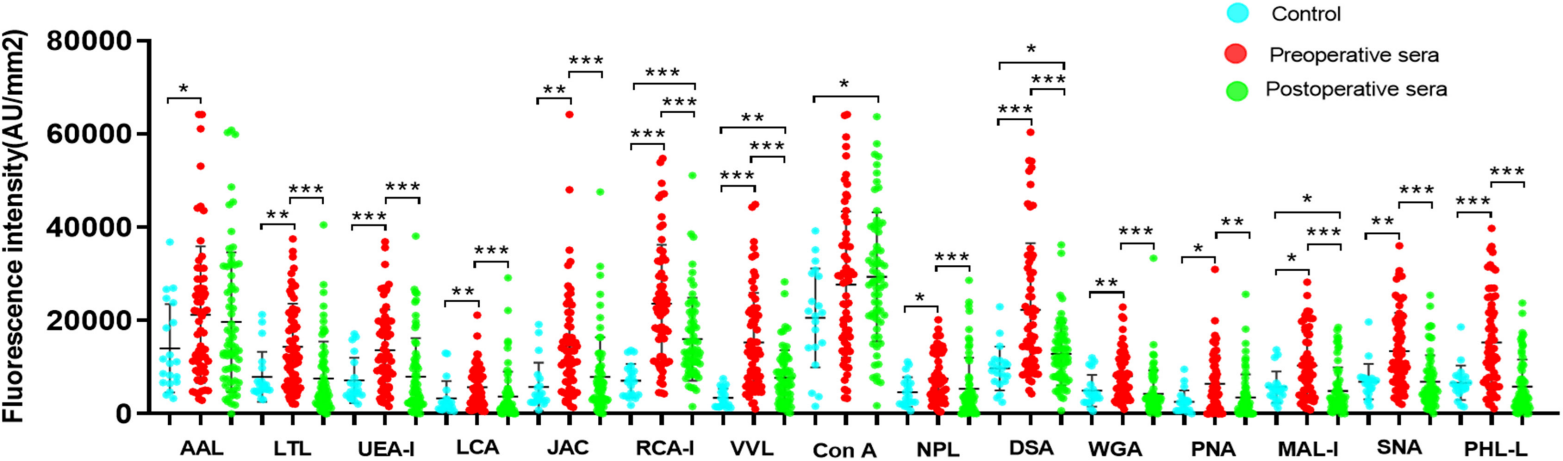
Comparisons of serum levels of lectin‐binding glycans between patients with HCC before operation, Day 7 after operation, and healthy individuals (means ± SD). *, *p* < 0.05; **, *p* < 0.01; ***, *p* < 0.001 (Mann–Whitney *U* test).

We evaluated the diagnostic efficacies for fifteen lectins and found that the lectins on the biochip bore moderate‐to‐good diagnostic efficacies by area under curve (AUC) values ranging 0.662–0.916 (Figure S1). Figure 1 showed that levels of lectin‐specific glycans in preoperative serum of the patients with HCC were significantly higher than those in the disease‐free individuals (*p* < 0.001 − *p* = 0.029), except ConA. On Day 7 after the operation, significantly decreased levels of lectin‐specific glycans, such as AAL, LTL, UEA‐I, LCA, JAC, NPL, WGA, PNA, SNA, and PHA‐L, in the serum of the patients were observed, which had no significant differences with those in the controls (*p* > 0.05). Expression of glycan‐recognizable ConA in postoperative serum was higher than level in the control (*p* = 0.016). In histochemical staining for NPL, stronger expression of NPL localized in the cytoplasm and membrane of most HCC cells and a few stromal cells in HCC tissues (Figure S2A), appearing brown‐yellow coarse spots. Weaker NPL staining was localized in the cytoplasm and membrane of sporadic hepatocytes in adjacent healthy hepatic tissues (Figure S2B).

### 3.2 Relationships of Serum Lectin‐Specific Glycans and Clinicopathological Characteristics of HCC Patients

**FIGURE 2:**
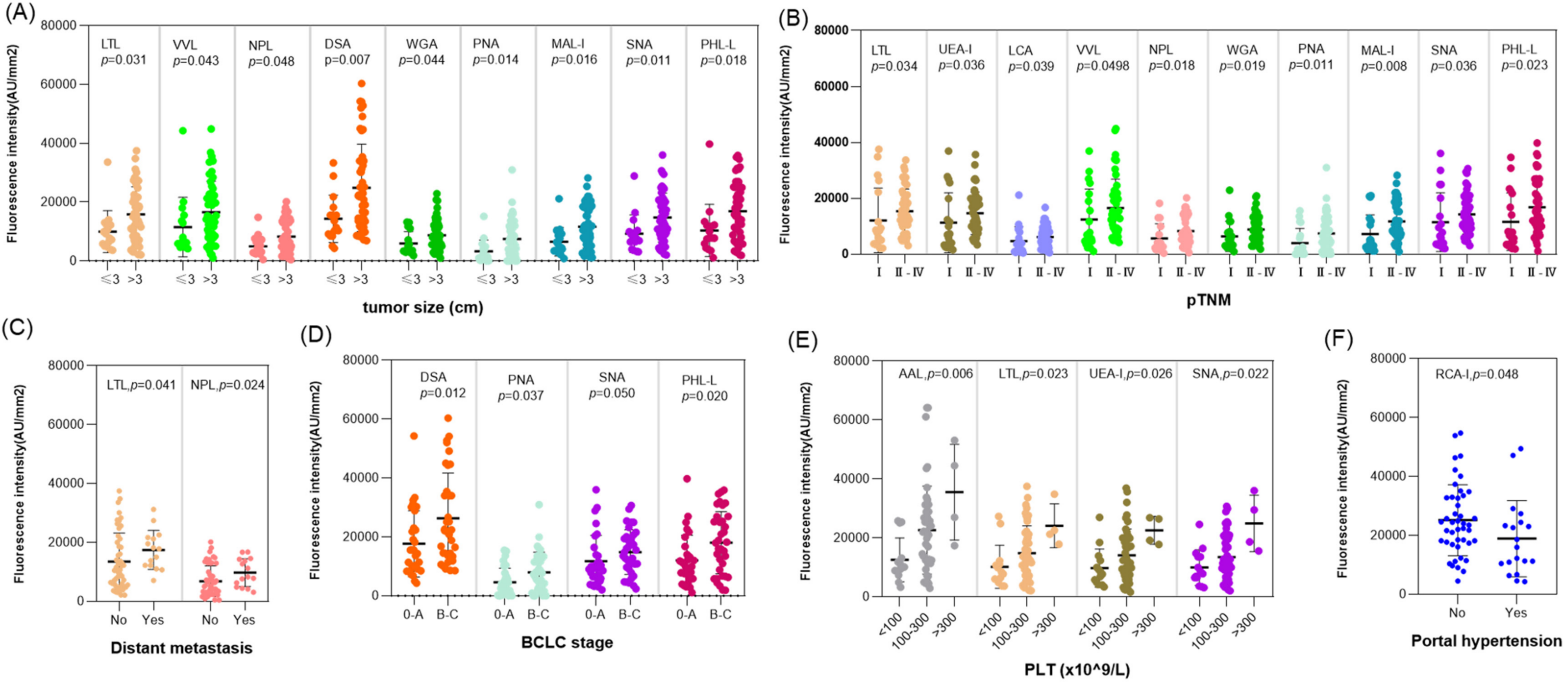
Comparison of levels of lectin‐binding glycans in preoperative serum and clinical characteristics in patients with HCC. (A) Tumor size (< 3 vs. ≥ 3 cm); (B) pTNM; (C) distant metastasis; (D) BCLC stage; (E) PLT (×10^9^/L); (F) portal hypertension (Mann–Whitney *U* and Kruskal–Wallis H test).

We analyzed relationships of serum lectin‐specific glycans and clinicopathological characteristics in the patients. We found that tumor size was significantly related to LTL, VVL, NPL, DSA, WGA, PNA, MAL‐I, SNA, and PHA‐L (Figure 2A); pTNM was related to LTL, UEA‐I, LCA, VVL, NPL, WGA, PNA, MAL‐I, SNA, and PHA‐L (Figure 2B); distant metastasis was related to LTL and NPL (Figure 2C); BCLC stage was related to DSA, PNA, SNA, and PHA‐L (Figure 2D); PLT was significantly related and was also positively correlated to AAL, LTL, UEA‐I, and SNA (Figure 2E); and PHT was related to RCA‐I, respectively (Figure 2F). Multivariate logistic analysis further indicated the tumor size was an independent factor impacting serum levels of glycan‐recognizable VVL, NPL, WGA, PNA, MAL‐I, and PHA‐L (*p* = 0.011–0.044); pTNM was significantly associated with LTL, UEA‐I, VVL, WGA, PNA, MAL‐I, SNA, and PHA‐L (*p* = 0.001–0.016); BCLC stage and PLT would be independent factors influencing expression of DSA (*p* = 0.024) and SNA (*p* = 0.050), respectively (Table 1).

**TABLE 1:** Multivariate logistic analysis of lectin‐binding glycans in preoperative serum and clinical characteristics in patients with HCC.

Parameters	LTL		UEA‐I		VVL	
	OR (95% CI)	*p*	OR (95% CI)	*p*	OR (95% CI)	*p*
PLT	NA		NA		—	
Tumor size	NA		—		6.11 (1.612–23.159)	0.008
pTNM	4.275 (1.314–13.912)	0.016	5.014 (1.499–16.775)	0.009	5.534 (1.537–19.926)	0.009
Distant metastasis	NA		—		—	
BCLC stage	—		—		—	

Parameters	NPL		DSA		WGA	
	OR (95% CI)	*p*	OR (95% CI)	*p*	OR (95% CI)	*p*
PLT	—		—		—	
Tumor size	8.710 (1.060–71.547)	0.044	NA		5.270 (1.471–18.885)	0.011
pTNM	NA		—		4.452 (1.328–14.926)	0.016
Distant metastasis	NA		—		—	
BCLC stage	—		3.696 (1.185–11.529)	0.024	—	

Parameters	PNA		MAL‐I		SNA	
	OR (95% CI)	*p*	OR (95% CI)	*p*	OR (95% CI)	*p*
PLT	—		—		3.349 (1.000–11.214)	0.050
Tumor size	4.628 (1.255–17.065)	0.021	5.448 (1.425–20.826)	0.013	NA	
pTNM	7.109 (2.044–24.729)	0.002	8.405 (2.333–30.284)	0.001	5.881 (1.733–19.955)	0.004
BCLC stage	NA		—		NA	

	PHA‐L	
	OR (95% CI)	*p*
Tumor size	3.730 (1.041–13.367)	0.043
pTNM	6.031 (1.795–20.261)	0.004
BCLC stage	NA	

*Note:* tumor size: < 3 vs. ≥ 3 cm; pTNM: I vs. II–IV; BCLC stage: 0–A vs. B–C.

On day 7 after operation, regardless of significantly decreased level of albumin (39.75 ± 4.08 g/L vs. 35.60 ± 4.19 g/L, *p* < 0.001) and elevated level of ALT (41.98 ± 40.75 U/L vs. 72.37 ± 41.43 U/L, *p* < 0.001) in the serum (Table S1), serum levels of the glycans did not display significant associations with main pathological characteristics and correlations to clinical chemical data of the HCC patients anymore. Rather we observed that differentiation of tumor significantly influenced serum glycan‐recognizable AAL (*p* = 0.039) and RCA‐I (*p* = 0.022, Figure S3A). Both alcohol intake (Figure S3B) and smoking habit (Figure S3C) were significantly related to levels of RCA‐I (*p* = 0.033 vs. *p* = 0.036), VVL (*p* = 0.015 vs. *p* = 0.002), and ConA (*p* = 0.011 vs. *p* = 0.005). Albumin and ABO type were significantly related to postoperative serum levels of VVL (*p* = 0.014, Figure S3D) and ConA (*p* = 0.004, Figure S3E). Multivariate logistic analysis indicated differentiation of tumor, albumin level, and ABO type of the patients presented independent influence on postoperative serum levels of RCA‐I (*p* = 0.024), VVL (*p* = 0.024), and ConA (*p* = 0.026), respectively (Table 2). Correlation analysis demonstrated that serum levels of the lectin‐specific glycans bore different correlations with PLT, ALT, AST, and AFP levels of patients with HCC before operation (Figure S4A) and on Day 7 after operation (Figure S4B).

**TABLE 2:** Multivariate logistic analysis of lectin‐binding glycans in postoperative serum and clinical characteristics in patients with HCC.^a^

Parameters	RCA‐I		VVL		ConA	
	OR (95% CI)	*p*	OR (95% CI)	*p*	OR (95% CI)	*p*
ABO type	—		—		1.623 (1.059–2.486)	0.026
Alcohol intake	NA		NA		NA	
Smoking	NA		NA		NA	
Albumin	—		12.000 (1.379–104.398)	0.024	—	
Differentiation	0.175 (0.038–0.799)	0.024	—		—	

^a^ On Day 7 after surgery.

### 3.3 Prognostic Values of Serum Lectin‐Specific Glycans for HCC Patients

**FIGURE 3:**
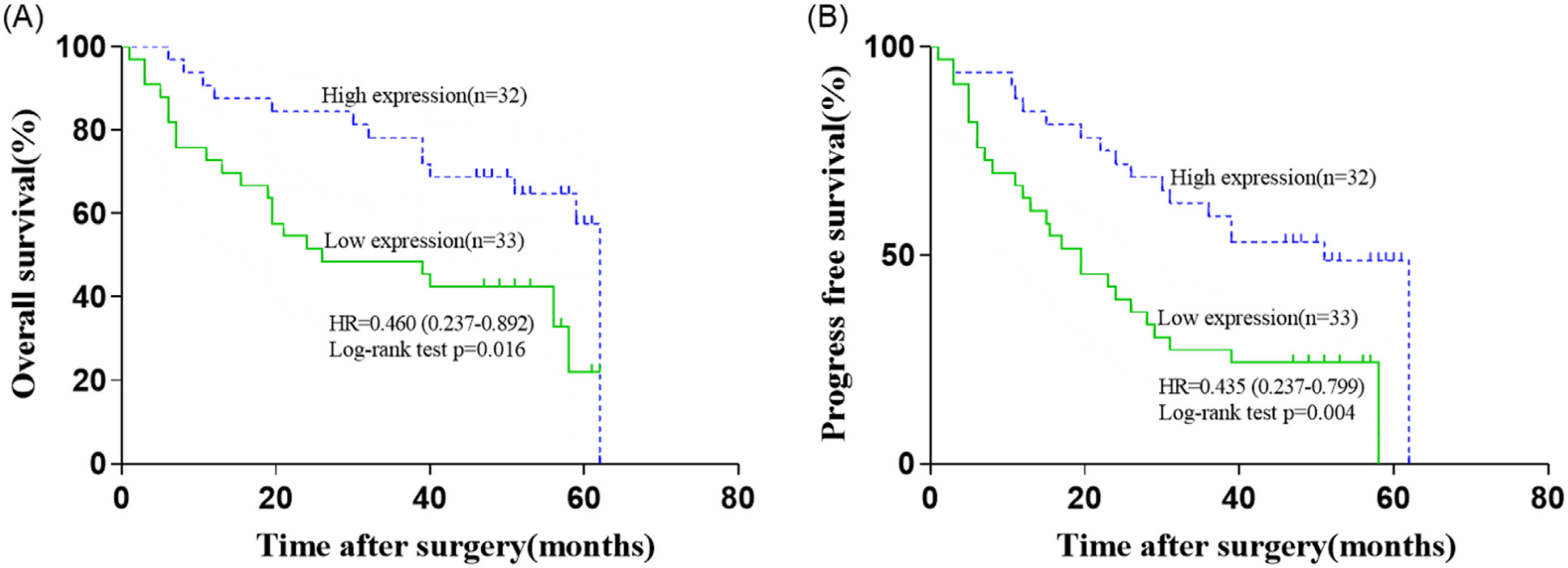
Kaplan–Meier curves of overall survival (OS, A) and progress‐free survival (PFS, B) in patients with HCC after surgery stratified according to VVL‐binding glycan levels in preoperative serum (Log‐rank test).

Finally, we carried out Kaplan–Meier analysis for evaluating the influence of the serum levels of lectin‐specific glycans on the outcome of the HCC patients. We observed that the HCC patients with high expression of VVL‐specific glycans in preoperative serum benefited a longer OS time (log‐rank test, *p* = 0.016, HR: 0.460, 95% CI: 0.237–0.892, Figure 3A) and a better PFS time (log‐rank test, *p* = 0.004, HR: 0.435, 95% CI: 0.237–0.799, Figure 3B). The overall five‐year survival rate of the HCC patients with high and low levels of VVL‐specific glycans in the serum was 56.25% and 33.33%, respectively. The five‐year PFS rates associated with high and low levels of serum VVL‐specific glycans were 43.75% and 21.21%, respectively.

## 4 Discussion

In this cohort study, we observed that levels of lectins‐binding glycans in the preoperative serum of the HCC patients were significantly higher than those in the healthy individuals (*p* < 0.001 − *p* = 0.029). Efficiently surgical excision of tumor mass led to quick and significant attenuations of the lectin signals in postoperative serum of the HHC patients. Stronger histochemical staining for NPL was localized in either tumor cells or some tumor‐stromal cells. This study and other investigation [13] indicated that increased levels of serum lectin‐binding glycans in the HCC patients would be produced from HCC tissues. The mechanisms remained unclear. Potential hypotheses would be attributable to either loss of cell polarity, altered adhesive properties [32], increase of enzymes involved in glycosylation [33], release or degradation via proteolysis [3], or impaired clearance of glycosylated proteins [34]. Increased expression of ConA‐binding glycans in postoperative serum would reflect the renovation process in liver tissue after surgery injury [35]. Our data indicated serum lectin‐specific glycans would objectively reflect the change of glycosylation status or release of glycans from tumor tissue per se or tumor‐stromal interaction in HCC.

We compared serum changes of lectin‐based glycans and clinicopathological characteristics of the HCC patients and found that changes of the lectins‐specific glycans were associated with progression and tumor invasion in the HCC patients. of which, terminal GalNAc glycotope, which is VVL, PNA, and SNA recognizable, participates in O‐glycosylation of proteins. Level of GalNAc glycotope in EGFR, MMP‐14, αV, β1, and β4 integrin positively correlates with invasive potential of tumor cell lines [36, 37, 38]. GlcNAc is an important glycotope binding to AAL, LTL, UEA‐I, LCA, RCA‐I, DSA, WGA, and MAL‐I. In this study, we observed that altered serum levels of glycotopes of GalNAc and GlcNAc were significantly associated with tumor size, pTNM, distant metastasis, and BCLC stage and PLT. More impressively, we found that DSA, PNA, SNA, and PHA‐I, binders of terminal GalNAc glycotope and triantennary and tetra‐antennary N‐glycans, were significantly related to BCLC stage in the HCC patients (*p* = 0.012 − *p* = 0.050). Although an additional investigation based on tissue microarray and lectin immunohistochemistry highlighted that NPA, ConA, GNA, and Calsepa, binding specificity to high‐mannose glycans, in HCC tissues were significantly increased in moderately‐differentiated HCC compared with those in well‐differentiated HCC [15], our lectin biochip data demonstrated that none of the lectin‐specific glycans in the preoperative serum of HCC patients were related to differentiation of HCC, and, however, that levels of AAL and RCA‐I in postoperative tumor‐free serum of the patients showed significant differences between well‐differentiated and moderate/poorly differentiated status of HCC (*p* = 0.022 and *p* = 0.039, respectively). The reasons remained unknown. The potential explanations would be that changes of serum AAL and RCA‐I levels in the tumor‐free patients after the surgical intervention may be attributable to status heterogeneity in individuals or differences in sample types or sources and measurement methodologies [15, 18]. In addition, our multivariate logistic analysis indicated the clinical characteristics of HCC patients, such as tumor size, pTNM, BCLC stage, and PLT would be independent factors influencing levels of serum glycans, respectively. Obviously, it would be of value that further attempts ask whether changes of structure patterns of serum glycosylated proteins bearing glycotopes of GalNAc, GlcNAc, and branching glycans would confer glycoproteins potentials in promoting invasion and enhancing aggressive progression of HCC patients. Previous studies revealed that age, diet, smoking, body mass, and blood lipid status are environmental factors influencing glycan levels [18]. In this study, we highlighted that the environmental factors were significantly related to the levels of serum lectin‐specific glycans in tumor‐free patients after operation.

Although a previous investigation based on tissue microarray, lectin histochemistry, and matrix‐assisted laser desorption/ionization mass spectrometry imaging (MALDI‐MSI) analysis demonstrated that increased levels of fucosylated glycoforms and tetra‐antennary glycan in HCC patients were associated with a reduction in survival time of HCC patients [13], in this study, we observed that the OS rate of the HCC patients with high serum level of VVL‐binding glycans was superior to those with low serum level. HCC patients with high‐expression level of VVL‐binding glycans benefited from a better outcome from surgical intervention than those with low‐expression level of VVL‐binding glycans in the serum. VVL specifically binds αGalNAc, GalNAcα3Gal, and Tn antigen [39]. Interpretation of biological effect of VVL‐recognizable glycosylated proteins on cancers remains augured. Previous investigations displayed that proteins bearing VVA‐binding carbohydrates implicate lymphatic metastasis in tumor cells [40]. Overexpression of core 1 β1,3‐galactosyltransferase (C1GALT1) significantly affected VVL binding ability to glycoproteins, thereby deteriorating progression and OS in HCC patients [41]. Other studies demonstrated that polypeptide N‐acetylgalactosaminyltransferase 5 (GalNAc‐T5)‐encoded glycophenotypes might possess tumor suppressor activity [42]. Elevated levels of GalNAc‐T5 led to an increased OS [43]. Obviously, the effect of GalNAc on the invasive potential of tumors seemed intricate, which would be related to types of cancer cells or glycosylations [3, 38]. Our study indicated that VVL‐recognizable glycotope αGalNAc was not only involved in metastasis and progression but also significantly influenced survival in patients with HCC. More detailed mechanisms concerning the effect of VVL‐related glycans on HCC would be highly expected.

Taken together, the measurement of serum glycans was a promising blood‐based test for HCC, which could reflect surgical outcomes in at‐risk patients and become valuable biomarkers in evaluating the progression of HCC patients.

## Author Contributions

**Sheng‐Sheng Liu:** formal analysis (equal), methodology (equal), software (equal), validation (equal), visualization (equal), writing – original draft (equal), writing – review and editing (equal). **Lei Ye:** methodology (equal), validation (equal), writing – review and editing (equal). **Qing‐Qing Dai:** data curation (equal), investigation (equal), resources (equal), validation (equal), writing – review and editing (equal). **Yi Gao:** formal analysis (equal), investigation (equal), methodology (equal), validation (equal), writing – review and editing (equal). **Guang‐Hou Chen:** data curation (equal), investigation (equal), resources (equal), validation (equal), writing – review and editing (equal). **Hong‐Chuan Zhao:** conceptualization (equal), funding acquisition (equal), project administration (equal), supervision (equal), validation (equal), writing – review and editing (equal). **Wei‐Dong Du:** conceptualization (equal), formal analysis (equal), funding acquisition (equal), methodology (equal), project administration (equal), supervision (equal), validation (equal), writing – review and editing (equal).

## Conflicts of Interest

The authors declare no conflicts of interest.

## Supporting information


**Table S1.** Clinical characteristics of patients with HCC.
**Table S2.** Lectins used, recognizable glycans, and potential indications.

**Figure S1.** Receiver operating characteristic (ROC) curves for discriminating individual lectins in HCC from disease‐free individuals.

**Figure S2.** Histochemical staining for Narcissus pseudonarcissus lectin (NPL) was conducted to reveal expression of NPL in HCC tissues. (A) Stronger expression of NPL in the cytoplasm and membrane of HCC cells (…) and stromal cells (…). (B) Weaker NPL staining was localized in the cytoplasm and membrane of sporadic hepatocytes (…) in adjacent healthy hepatic tissues. Original magnification: ×200.

**Figure S3.** Comparison of serum levels of lectin‐binding glycan between postoperative serum and clinical characteristics of patients with HCC. (A) differentiation; (B) alcohol intake; (C) smoking; (D) albumin; (E) ABO type (Mann–Whitney *U* and Kruskal–Wallis *H* test).

**FIGURE S4.** Correlation matrix of serum glycan‐binding lectins and data of clinical laboratory tests in patients with HCC before operation (A) and on Day 7 after operation (B) (Spearman’s test).

## Data Availability

The data that support the findings of this study are available from the corresponding authors upon reasonable request.

## References

[1] H. Sung , J. Ferlay , R. L. Siegel , et al., “Global Cancer Statistics 2020: GLOBOCAN Estimates of Incidence and Mortality Worldwide for 36 Cancers in 185 Countries,” CA: A Cancer Journal for Clinicians 71, no. 3 (2021): 209–249, 10.3322/caac.21660. 33538338

[2] H. Rumgay , J. Ferlay , C. de Martel , et al., “Global, Regional and National Burden of Primary Liver Cancer by Subtype,” European Journal of Cancer 161 (2022): 108–118, 10.1016/j.ejca.2021.11.023. 34942552

[3] K. Ohtsubo and J. D. Marth , “Glycosylation in Cellular Mechanisms of Health and Disease,” Cell 126, no. 5 (2006): 855–867, 10.1016/j.cell.2006.08.019. 16959566

[4] C. Reily , T. J. Stewart , M. B. Renfrow , and J. Novak , “Glycosylation in Health and Disease,” Nature Reviews. Nephrology 15, no. 6 (2019): 346–366, 10.1038/s41581-019-0129-4. 30858582 PMC6590709

[5] X. Verhelst , A. M. Dias , J. F. Colombel , et al., “Protein Glycosylation as a Diagnostic and Prognostic Marker of Chronic Inflammatory Gastrointestinal and Liver Diseases,” Gastroenterology 158, no. 1 (2020): 95–110, 10.1053/j.gastro.2019.08.060. 31626754

[6] A. Mehta , H. Herrera , and T. Block , “Glycosylation and Liver Cancer,” Advances in Cancer Research 126 (2015): 257–279, 10.1016/bs.acr.2014.11.005. 25727150 PMC4634841

[7] S. S. Pinho and C. A. Reis , “Glycosylation in Cancer: Mechanisms and Clinical Implications,” Nature Reviews. Cancer 15, no. 9 (2015): 540–555, 10.1038/nrc3982. 26289314

[8] R. R. Drake , “Glycosylation and Cancer: Moving Glycomics to the Forefront,” Advances in Cancer Research 126 (2015): 1–10, 10.1016/bs.acr.2014.12.002. 25727144

[9] S. M. Kremsreiter , A. H. Kroell , K. Weinberger , and H. Boehm , “Glycan‐Lectin Interactions in Cancer and Viral Infections and How to Disrupt Them,” International Journal of Molecular Sciences 22, no. 19 (2021): 10577, 10.3390/ijms221910577. 34638920 PMC8508825

[10] N. Very , T. Lefebvre , and I. El Yazidi‐Belkoura , “Drug Resistance Related to Aberrant Glycosylation in Colorectal Cancer,” Oncotarget 9, no. 1 (2018): 1380–1402, 10.18632/oncotarget.22377. 29416702 PMC5787446

[11] J. C. de Freitas Junior and J. A. Morgado‐Diaz , “The Role of N‐Glycans in Colorectal Cancer Progression: Potential Biomarkers and Therapeutic Applications,” Oncotarget 7, no. 15 (2016): 19395–19413, 10.18632/oncotarget.6283. 26539643 PMC4991391

[12] C. Bull , M. H. den Brok , and G. J. Adema , “Sweet Escape: Sialic Acids in Tumor Immune Evasion,” Biochimica et Biophysica Acta 1846, no. 1 (2014): 238–246, 10.1016/j.bbcan.2014.07.005. 25026312

[13] C. A. West , M. Wang , H. Herrera , et al., “N‐Linked Glycan Branching and Fucosylation Are Increased Directly in Hcc Tissue as Determined Through In Situ Glycan Imaging,” Journal of Proteome Research 17, no. 10 (2018): 3454–3462, 10.1021/acs.jproteome.8b00323. 30110170 PMC6784322

[14] A. DelaCourt , A. Black , P. Angel , et al., “N‐Glycosylation Patterns Correlate With Hepatocellular Carcinoma Genetic Subtypes,” Molecular Cancer Research 19, no. 11 (2021): 1868–1877, 10.1158/1541-7786.MCR-21-0348. 34380744 PMC8802325

[15] H. Takayama , M. Ohta , Y. Iwashita , et al., “Altered Glycosylation Associated With Dedifferentiation of Hepatocellular Carcinoma: A Lectin Microarray‐Based Study,” BMC Cancer 20, no. 1 (2020): 192, 10.1186/s12885-020-6699-5. 32143591 PMC7060603

[16] C. Sun , P. Chen , Q. Chen , et al., “Serum Paraoxonase 1 Heteroplasmon, a Fucosylated, and Sialylated Glycoprotein in Distinguishing Early Hepatocellular Carcinoma From Liver Cirrhosis Patients,” Acta Biochimica et Biophysica Sinica Shanghai 44, no. 9 (2012): 765–773, 10.1093/abbs/gms055. 22751611

[17] M. Wang , M. Sanda , M. A. Comunale , et al., “Changes in the Glycosylation of Kininogen and the Development of a Kininogen‐Based Algorithm for the Early Detection of HCC,” Cancer Epidemiology, Biomarkers & Prevention 26, no. 5 (2017): 795–803, 10.1158/1055-9965.EPI-16-0974. PMC5759760 28223431

[18] S. Zhang , X. Cao , Q. Gao , and Y. Liu , “Protein Glycosylation in Viral Hepatitis‐Related HCC: Characterization of Heterogeneity, Biological Roles, and Clinical Implications,” Cancer Letters 406 (2017): 64–70, 10.1016/j.canlet.2017.07.026. 28789967

[19] Y. S. Fang , Q. Wu , H. C. Zhao , et al., “Do Combined Assays of Serum AFP, AFP‐L3, DCP, GP73, and DKK‐1 Efficiently Improve the Clinical Values of Biomarkers in Decision‐Making for Hepatocellular Carcinoma? A Meta‐Analysis,” Expert Review of Gastroenterology & Hepatology 15, no. 9 (2021): 1065–1076, 10.1080/17474124.2021.1900731. 33691550

[20] K. Dang , W. Zhang , S. Jiang , X. Lin , and A. Qian , “Application of Lectin Microarrays for Biomarker Discovery,” ChemistryOpen 9, no. 3 (2020): 285–300, 10.1002/open.201900326. 32154049 PMC7050261

[21] H. Du , H. Yu , F. Yang , and Z. Li , “Comprehensive Analysis of Glycosphingolipid Glycans by Lectin Microarrays and MALDI‐TOF Mass Spectrometry,” Nature Protocols 16, no. 7 (2021): 3470–3491, 10.1038/s41596-021-00544-y. 34099941

[22] J. Hirabayashi , M. Yamada , A. Kuno , and H. Tateno , “Lectin Microarrays: Concept, Principle and Applications,” Chemical Society Reviews 42, no. 10 (2013): 4443–4458, 10.1039/c3cs35419a. 23443201

[23] Y. Qin , Y. Zhong , T. Ma , et al., “Alteration of Liver Glycopatterns During Cirrhosis and Tumor Progression Induced by HBV,” Glycoconjugate Journal 33, no. 2 (2016): 125–136, 10.1007/s10719-015-9645-z. 26833199

[24] C. B. Satala , I. Jung , L. Kobori , et al., “Benefits of the 8th American Joint Committee on Cancer System for Hepatocellular Carcinoma Staging,” Journal of Gastrointestinal Cancer 52, no. 1 (2021): 243–248, 10.1007/s12029-020-00394-z. 32173767

[25] H. Yan , X. Wang , X. Liu , et al., “The Survival Strength of Younger Patients in BCLC Stage 0‐B of Hepatocellular Carcinoma: Basing on Competing Risk Model,” BMC Cancer 22, no. 1 (2022): 185, 10.1186/s12885-022-09293-x. 35180841 PMC8855543

[26] D. Bojar , L. Meche , G. Meng , et al., “A Useful Guide to Lectin Binding: Machine‐Learning Directed Annotation of 57 Unique Lectin Specificities,” ACS Chemical Biology 17, no. 11 (2022): 2993–3012, 10.1021/acschembio.1c00689. 35084820 PMC9679999

[27] Y. Xie , Y. Sheng , Q. Li , S. Ju , J. Reyes , and C. B. Lebrilla , “Determination of the Glycoprotein Specificity of Lectins on Cell Membranes Through Oxidative Proteomics,” Chemical Science 11, no. 35 (2020): 9501–9512, 10.1039/D0SC04199H. 34094216 PMC8162070

[28] L. Ye , N.‐L. Huang , X.‐L. Ma , M. Schneider , X.‐J. Huang , and W.‐D. Du , “Establishment of N‐Succinimidyl 4‐(Maleimidomethyl) Cyclohexanecarboxylate (SMCC) Modified Biochip Enabling Concurrent Detection of Serum Infectious Antibodies in Neuroborreliosis,” Biosensors and Bioelectronics 78 (2016): 404–410, 10.1016/j.bios.2015.11.050. 26655180

[29] Y. Gao , S.‐G. Li , Q. Liu , et al., “Establishment of a 1, 4, 7, 10‐Tetraazacyclododecane‐1, 4, 7, 10‐Tetraacetic Acid Mono‐N‐Hydroxysuccinimide Ester (DOTA‐NHS‐Ester) Based Lectin Microarray for Efficiently Detecting Serum Glycans in Gastric Cancers,” Analytical Biochemistry 597 (2020): 113686, 10.1016/j.ab.2020.113686. 32156505

[30] S. Li , C. Mo , Q. Peng , et al., “Cell Surface Glycan Alterations in Epithelial Mesenchymal Transition Process of Huh7 Hepatocellular Carcinoma Cell,” PLoS One 8, no. 8 (2013): e71273, 10.1371/journal.pone.0071273. 23977005 PMC3748092

[31] S. S. Liu , Y. Gao , S. P. Yin , et al., “Expression of Narcissus Pseudonarcissus Lectin and Mannose Receptor Positive Macrophages Predict Progression and Prognosis of Patients With Gastric Cancer,” Translational Cancer Research 9, no. 10 (2020): 5979–5993, 10.21037/tcr-20-1459. 35117210 PMC8798769

[32] Y. Cao , H. Chang , L. Li , R. C. Cheng , and X. N. Fan , “Alteration of Adhesion Molecule Expression and Cellular Polarity in Hepatocellular Carcinoma,” Histopathology 51, no. 4 (2007): 528–538, 10.1111/j.1365-2559.2007.02820.x. 17880531

[33] K. Moriwaki , K. Noda , T. Nakagawa , et al., “A High Expression of GDP‐Fucose Transporter in Hepatocellular Carcinoma Is a Key Factor for Increases in Fucosylation,” Glycobiology 17, no. 12 (2007): 1311–1320, 10.1093/glycob/cwm094. 17884843

[34] J. B. Burgess , J. U. Baenziger , and W. R. Brown , “Abnormal Surface Distribution of the Human Asialoglycoprotein Receptor in Cirrhosis,” Hepatology 15, no. 4 (1992): 702–706, 10.1002/hep.1840150425. 1372583

[35] Z. Wei , L. Huang , L. Cui , and X. Zhu , “Mannose: Good Player and Assister in Pharmacotherapy,” Biomedicine & Pharmacotherapy 129 (2020): 110420, 10.1016/j.biopha.2020.110420. 32563989

[36] S. R. Stateva and A. Villalobo , “O‐GlcNAcylation of the Human Epidermal Growth Factor Receptor,” Organic & Biomolecular Chemistry 13, no. 30 (2015): 8196–8204, 10.1039/c5ob00443h. 26108188

[37] C. H. Liu , R. H. Hu , M. J. Huang , et al., “C1GALT1 Promotes Invasive Phenotypes of Hepatocellular Carcinoma Cells by Modulating Integrin beta1 Glycosylation and Activity,” PLoS One 9, no. 8 (2014): e94995, 10.1371/journal.pone.0094995. 25089569 PMC4121071

[38] E. Khosrowabadi , T. Wenta , S. Keskitalo , A. Manninen , and S. Kellokumpu , “Altered Glycosylation of Several Metastasis‐Associated Glycoproteins With Terminal GalNAc Defines the Highly Invasive Cancer Cell Phenotype,” Oncotarget 13 (2022): 73–89, 10.18632/oncotarget.28167. 35028012 PMC8751650

[39] K. Yamashita , A. Kuno , A. Matsuda , et al., “Lectin Microarray Technology Identifies Specific Lectins Related to Lymph Node Metastasis of Advanced Gastric Cancer,” Gastric Cancer 19, no. 2 (2016): 531–542, 10.1007/s10120-015-0491-2. 25840959

[40] T. Kawaguchi , H. Takazawa , S. Imai , et al., “Expression of Vicia Villosa Agglutinin (VVA)‐Binding Glycoprotein in Primary Breast Cancer Cells in Relation to Lymphatic Metastasis: Is Atypical MUC1 Bearing Tn Antigen a Receptor of VVA?,” Breast Cancer Research and Treatment 98, no. 1 (2006): 31–43, 10.1007/s10549-005-9115-6. 16752227

[41] Y. M. Wu , C. H. Liu , M. J. Huang , et al., “C1GALT1 Enhances Proliferation of Hepatocellular Carcinoma Cells via Modulating MET Glycosylation and Dimerization,” Cancer Research 73, no. 17 (2013): 5580–5590, 10.1158/0008-5472.CAN-13-0869. 23832667

[42] T. Caffrey , S. Sagar , D. Thomas , M. E. Lewallen , M. A. Hollingsworth , and P. Radhakrishnan , “The Glycoprotein Mucin‐1 Negatively Regulates GalNAc Transferase 5 Expression in Pancreatic Cancer,” FEBS Letters 593, no. 19 (2019): 2751–2761, 10.1002/1873-3468.13532. 31283009 PMC7048170

[43] H. He , Z. Shen , H. Zhang , et al., “Clinical Significance of Polypeptide N‐Acetylgalactosaminyl Transferase‐5 (GalNAc‐T5) Expression in Patients With Gastric Cancer,” British Journal of Cancer 110, no. 8 (2014): 2021–2029, 10.1038/bjc.2014.93. 24619076 PMC3992513

