# Supplementary material for: Preoperative Serum Glycan Levels Reflect Progression of Patients With Hepatocellular Carcinoma
Source: Cancer Med. 2024 Oct 9;13(19):e70285. doi: 10.1002/cam4.70285 (PMC11462596; doi:10.1002/cam4.70285)
Supplement: Supplementary file 1 — Table S1. Clinical characteristics of patients with HCC. Table S2. Lectins used, recognizable glycans, and potential indications. [file CAM4-13-e70285-s004.docx]

SUPPLEMENTARY TABLE 1 Clinical characteristics of patients with HCC.

| Variables | Preoperative HCC | |  | Postoperative HCC* | |  |
| --- | --- | --- | --- | --- | --- | --- |
|  | N | Case |  | N | Case | *p*-values |
| Gender (n，%) | 65 |  |  |  |  |  |
| male |  | 57 (87.7) |  |  |  |  |
| female |  | 8 (12.3) |  |  |  |  |
| Age (years) | 65 | 57.05±10.66 |  |  |  |  |
| ≥50 (n, %) |  | 47（72.3） |  |  |  |  |
| <50 (n, %) |  | 18（27.7） |  |  |  |  |
| ABO type (n, %) | 65 |  |  |  |  |  |
| A |  | 21（32.8） |  |  |  |  |
| B |  | 16（25.0） |  |  |  |  |
| AB |  | 4（6.3） |  |  |  |  |
| O |  | 23（35.9） |  |  |  |  |
| BMI | 64 | 22.19±2.84 |  |  |  |  |
| ≥25 (n, %) |  | 12（18.8） |  |  |  |  |
| <25 (n, %) |  | 52（81.2） |  |  |  |  |
| Alcohol intake (n, %) | 65 |  |  |  |  |  |
| Yes |  | 11 (16.9) |  |  |  |  |
| No |  | 54 (83.1) |  |  |  |  |
| Smoking (n, %) | 65 |  |  |  |  |  |
| Yes |  | 13 (20.0) |  |  |  |  |
| No |  | 52 (80) |  |  |  |  |
| PLT (x10^9/L) | 65 | 158.92±87.33 |  | 64 | 168.84±83.34 | 0.593 |
| <100 (n, %) |  | 17（21.5） |  |  | 15（23.4） |  |
| 100-300 (n, %) |  | 47（72.3） |  |  | 45（70.3） |  |
| >300 (n, %) |  | 4（6.2） |  |  | 4（6.3） |  |
| Albumin (g/L) | 65 | 39.75±4.08 |  | 65 | 35.60±4.19 | ＜0.001 |
| ≥40 (n, %) |  | 28（43.1） |  |  | 8（12.3） |  |
| <40 (n, %) |  | 37 (46.9) |  |  |  |  |
| Total bilirubin (μmol/L) | 65 | 16.46±8.40 |  | 65 | 19.35±18.68 | 0.905 |
| ≥17.1 (n, %) |  | 24（36.9） |  |  | 24（36.9） |  |
| <17.0 (n, %) |  | 41 (63.1) |  |  |  |  |
| ALT (U/L) | 64 | 41.98±40.75 |  | 64 | 72.37±41.43 | ＜0.001 |
| <40 (n, %) |  | 17（26.6） |  |  | 18（27.7） |  |
| ≥40 (n, %) |  | 47（73.4） |  |  | 47（72.3） |  |
| AST (U/L) | 64 | 44.39±37.28 |  | 64 | 42.27±27.96 | 0.901 |
| <40 (n, %) |  | 39（60.9） |  |  | 40（61.5） |  |
| ≥40 (n, %) |  | 25（39.1） |  |  | 25（38.5） |  |
| AFP (ng/mL) | 61 | 475.47±569.53 |  | 45 | 435.81±623.64 | 0.746 |
| <400 (n, %) |  | 37（60.7） |  |  | 31（68.9） |  |
| ≥400 (n, %) |  | 24（39.3） |  |  | 14（31.1） |  |
| HBsAg (n, %) | 65 |  |  |  |  |  |
| Yes |  | 50 (65.8) |  |  |  |  |
| No |  | 15 (34.2) |  |  |  |  |
| LC (n, %) | 65 |  |  |  |  |  |
| Yes |  | 49 (75.4) |  |  |  |  |
| No |  | 16 (24.6) |  |  |  |  |
| PHT (n, %) | 63 |  |  |  |  |  |
| Yes |  | 19 (30.2) |  |  |  |  |
| No |  | 44 (69.8) |  |  |  |  |
| Tumor location （n, %) | 65 |  |  |  |  |  |
| right lobe |  | 43（66.2） |  |  |  |  |
| left lobe |  | 22（33.8） |  |  |  |  |
| Tumor size (cm) | 65 | 6.57±4.32 |  |  |  |  |
| <3 (n, %) |  | 16（24.6） |  |  |  |  |
| ≥3 (n, %) |  | 49（75.4） |  |  |  |  |
| Differentiation (n, %) | 65 |  |  |  |  |  |
| well |  | 12(18.5) |  |  |  |  |
| moderate |  | 40(61.5) |  |  |  |  |
| poorly |  | 13(20.0) |  |  |  |  |
| LVI (n, %) | 63 |  |  |  |  |  |
| Yes |  | 38 (60.3) |  |  |  |  |
| No |  | 25 (39.7) |  |  |  |  |
| Capsule invasion (n, %) | 63 |  |  |  |  |  |
| Yes |  | 18 (28.6) |  |  |  |  |
| No |  | 45 (71.4) |  |  |  |  |
| Distant metastasis (n, %) | 65 |  |  |  |  |  |
| Yes |  | 15 (23.1) |  |  |  |  |
| No |  | 50 (76.9) |  |  |  |  |
| pTNM (n, %) | 65 |  |  |  |  |  |
| I |  | 19 (29.2) |  |  |  |  |
| II |  | 29 (44.6) |  |  |  |  |
| III |  | 1(1.5) |  |  |  |  |
| IV |  | 16 (24.6) |  |  |  |  |
| Child-Turcotte-Pugh-Score (n, %) | 62 |  |  |  |  |  |
| A |  | 50 (80.6) |  |  |  |  |
| B |  | 12（19.4） |  |  |  |  |
| C |  | 0（0） |  |  |  |  |
| BCLC stage (n, %) | 65 |  |  |  |  |  |
| 0 |  | 4 (6.2) |  |  |  |  |
| A |  | 26 (40.0) |  |  |  |  |
| B |  | 32 (49.2) |  |  |  |  |
| C |  | 3 (4.6) |  |  |  |  |

*: on day 7 after operation

Abbreviations: N, number; HCC, hepatocellular carcinoma；BMI, body mass index; PLT, platelet count; ALT, alanine aminotransferase; AST, aspartate aminotransferase; AFP, alpha fetoprotein; HBsAg, HBV surface antigen; LC, liver cirrhosis; PHT, portal hypertension; LVI, lymphovascular invasion; pTNM, tumor-node-metastasis; BCLC stage, Barcelona Clinic Liver Cancer.

SUPPLEMENTARY TABLE 2 Lectins used, recognizable glycans and potential indications.

| Name | Abbreviation | Monosaccharide specificity | Preferred glycan structure (terminal epitope)^1, 2^ | Indications |
| --- | --- | --- | --- | --- |
| Aleuria aurantia lectinn | AAL | Fuc | Fucα6GlcNAc (core Fuc), Fucα3(Galβ4) GlcNAc (Lex) |  |
| Lotus tetragonolobus lectin | LTL | Fuc | Fucα3(Galβ4) GlcNAc (Lex), Fucα2Galβ4GlcNAc (H-type 2) |  |
| Ulex europaeus agglutinin | UEA-I | Fuc | Fucα2Galβ4GlcNAc (H-type 2) | Early/ advanced^3^ |
| Lens culinaris agglutinin | LCA | Fuc/Man | Fucα6GlcNAc, High-Man | Metastasis^4^ |
| Concanavalin A | Con A | Man | High-Man, including Manα6(Manα3) Man | Differentiation^5^ |
| Narcissus pseudonarcissus lectin | NPL | Man | High-Man, including Manα6Man | Differentiation^5^ |
| Jacalin | JAC | Gal | Galβ3GalNAc, αGalNAc (6O-unsubstituted) |  |
| Ricinus communis agglutinin-I | RCA-I | Gal | Galβ4GlcNAc |  |
| Peanut agglutinin | PNA | Gal | Galβ3GalNAc |  |
| Vicia villosa lectin | VVL | GalNAc | GalNAc and GalNAcα-Ser/Thr (Tn) |  |
| Datura stramonium agglutinin | DSA | GlcNAc | (GlcNAcβ4) n, triantennary, tetra-antennary N-glycans | Metastasis^4^ |
| Wheat germ agglutinin | WGA | GlcNAc | (GlcNAc)n and multivalent Sia | Metastasis^4, 6^ |
| Maackia amurensis lectin-I | MAL-I | Sia | Sia2-3Galβ1-4GlcNAc and Galβ1,4GlcNAc | Metastasis^4^ |
| Sambucus nigra lectin | SNA | Sia | Siaα2-6Gal/GalNAc |  |
| Phaseolus vulgaris Leucoagglutinin | PHA-L | Complex | β-1, 6 branching tri/tetra-antennary complex-type N-glycan | Metastasis^4^ |

Notes: Fuc, fucose; Gal, galactose; Man, mannose; GalNAc, N-Acetyl-D-galactosamine; GlcNAc, N-acetyl-D-glucosamine; Sia, sialic acid

**REFERENCES**

1 Xie Y, Sheng Y, Li Q, Ju S, Reyes J, Lebrilla CB. Determination of the glycoprotein specificity of lectins on cell membranes through oxidative proteomics. Chemical science. 2020;11(35):9501-12.doi:10.1039/D0SC04199H

2 Bojar D, Meche L, Meng G, Eng W, Smith DF, Cummings RD, et al. A useful guide to lectin binding: machine-learning directed annotation of 57 unique lectin specificities. ACS Chemical Biology. 2022;17(11):2993-3012.doi:10.1021/acschembio.1c00689

3 Sakamoto M, Ino Y, Fujii T, Hirohashi S. Phenotype changes in tumor vessels associated with the progression of hepatocellular carcinoma. Japanese journal of clinical oncology. 1993;23(2):98-104.doi:10.1093/oxfordjournals.jjco.a039623

4 Liu T, Shang S, Li W, Qin X, Sun L, Zhang S, et al. Assessment of Hepatocellular Carcinoma Metastasis Glycobiomarkers Using Advanced Quantitative N-glycoproteome Analysis. Front Physiol. 2017;8:472.doi:10.3389/fphys.2017.00472

5 Takayama H, Ohta M, Iwashita Y, Uchida H, Shitomi Y, Yada K, et al. Altered glycosylation associated with dedifferentiation of hepatocellular carcinoma: a lectin microarray-based study. BMC Cancer. 2020;20(1):192.doi:10.1186/s12885-020-6699-5

6 Qin X, Chen Q, Sun C, Wang C, Peng Q, Xie L, et al. High-throughput screening of tumor metastatic-related differential glycoprotein in hepatocellular carcinoma by iTRAQ combines lectin-related techniques. Med Oncol. 2013;30(1):420.doi:10.1007/s12032-012-0420-8
