# Supplementary figures and images for: Preoperative Serum Glycan Levels Reflect Progression of Patients With Hepatocellular Carcinoma
Source: Cancer Med. 2024 Oct 9;13(19):e70285. doi: 10.1002/cam4.70285 (PMC11462596; doi:10.1002/cam4.70285)

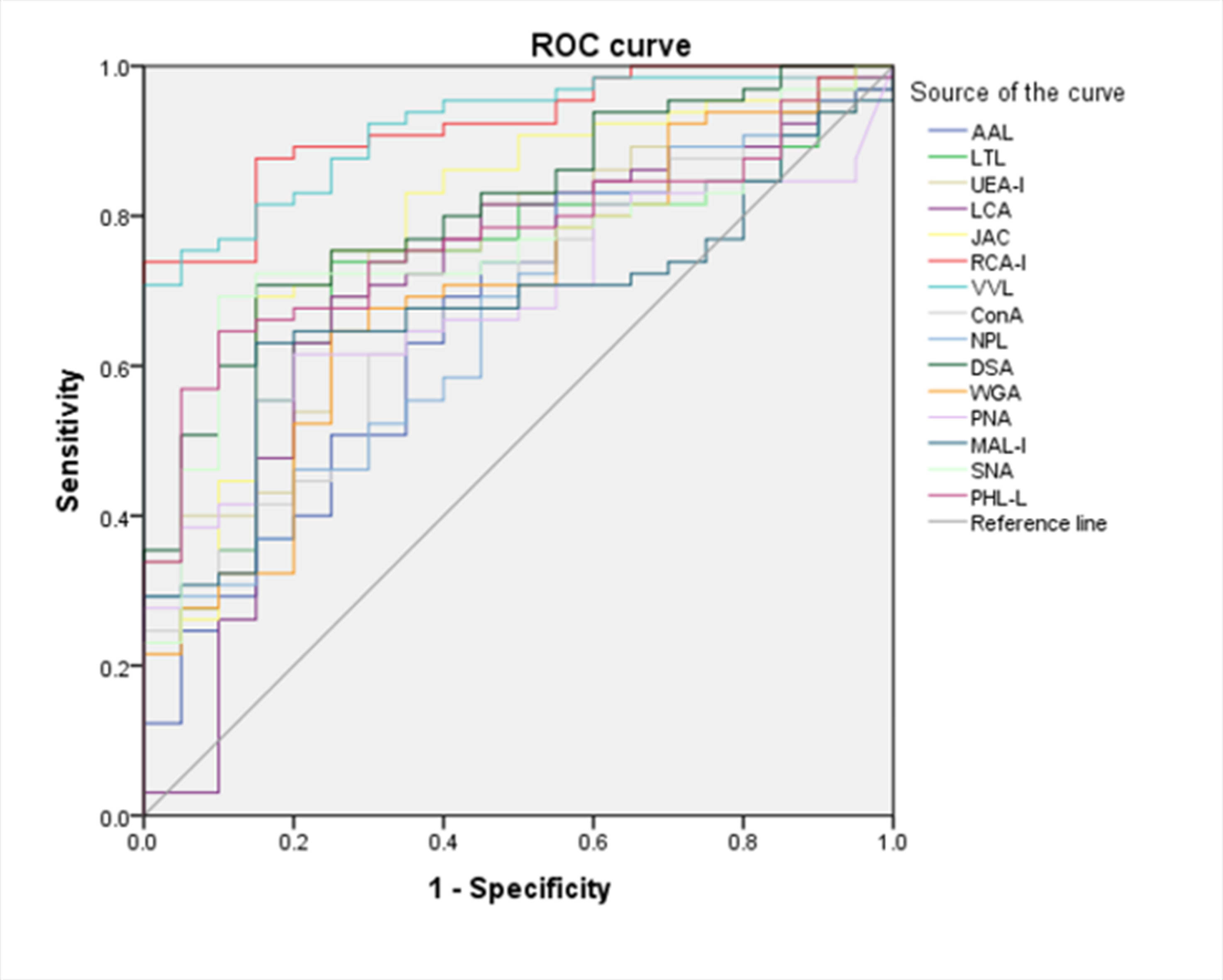

Supplement: Supplementary file 2 — Figure S1. Receiver operating characteristic (ROC) curves for discriminating individual lectins in HCC from disease‐free individuals. [file CAM4-13-e70285-s003.tif]

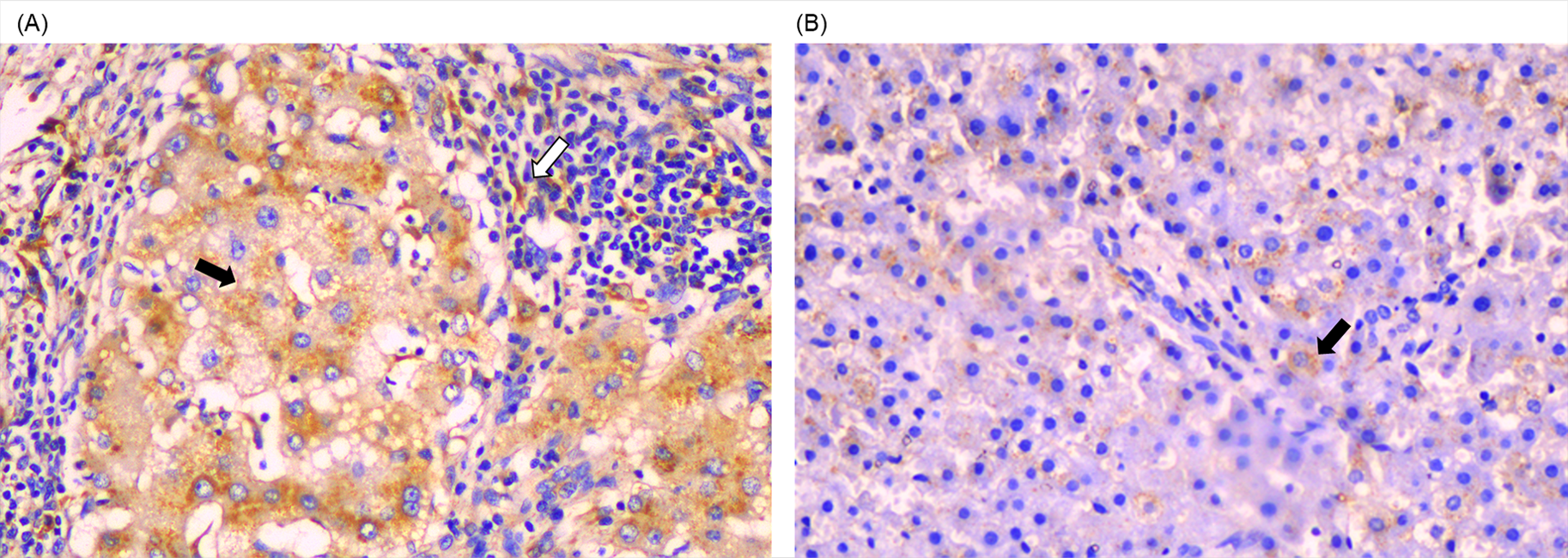

Supplement: Supplementary file 3 — Figure S2. Histochemical staining for Narcissus pseudonarcissus lectin (NPL) was conducted to reveal expression of NPL in HCC tissues. (A) Stronger expression of NPL in the cytoplasm and membrane of HCC cells (…) and stromal cells (…). (B) Weaker NPL staining was localized in the cytoplasm and membrane of sporadic hepatocytes (…) in adjacent healthy hepatic tissues. Original magnification: ×200. [file CAM4-13-e70285-s001.tif]

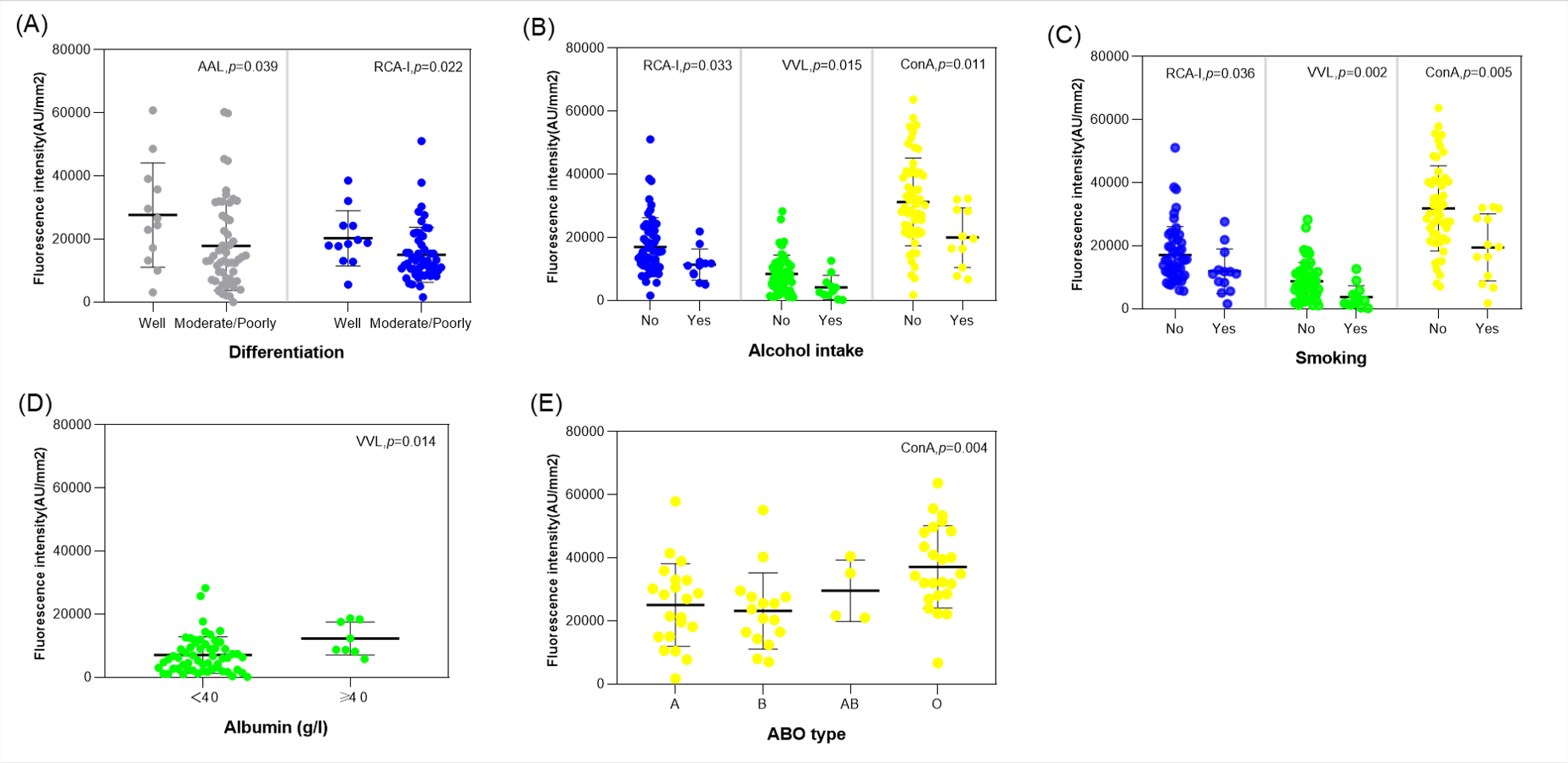

Supplement: Supplementary file 4 — Figure S3. Comparison of serum levels of lectin‐binding glycan between postoperative serum and clinical characteristics of patients with HCC. (A) differentiation; (B) alcohol intake; (C) smoking; (D) albumin; (E) ABO type (Mann–Whitney U and Kruskal–Wallis H test). [file CAM4-13-e70285-s005.tif]

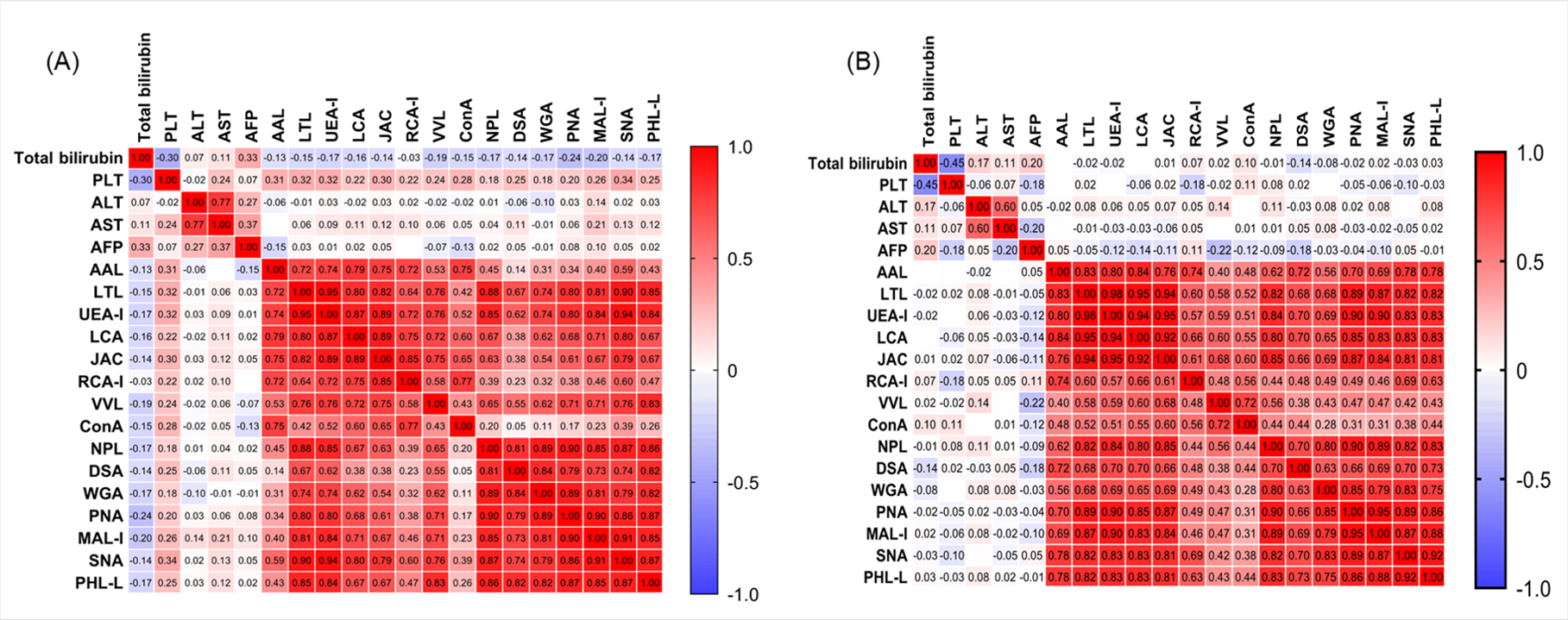

Supplement: Supplementary file 5 — FIGURE S4. Correlation matrix of serum glycan‐binding lectins and data of clinical laboratory tests in patients with HCC before operation (A) and on Day 7 after operation (B) (Spearman’s test). [file CAM4-13-e70285-s002.tif]
